# Diagnosis and treatment of bullous pemphigoid that developed twice after total knee replacement arthroplasty: a case report

**DOI:** 10.1186/s12891-021-04000-6

**Published:** 2021-01-28

**Authors:** Yong-Beom Kim, Hyung-Suk Choi, Hyung-Ki Cho, Gi-Won Seo

**Affiliations:** 1 Department of Orthopaedic Surgery, Soonchunhyang University Hospital Seoul, Seoul, South Korea; 2 Department of Orthopaedic Surgery, Soonchunhyang University Hospital Gumi, 179, 1gongdan-ro, Gumi, Gyeongsangbuk-do 39371 South Korea

**Keywords:** Bullous pemphigoid, Generalized bullous pemphigoid, Total knee replacement arthroplasty, Postoperative course

## Abstract

**Background:**

Total knee replacement arthroplasty (TKA) is frequently performed in South Korea. Simple swelling-associated blistering around the periphery of the operative wound is a well-known adverse effect. However, in rare cases, the blisters are bullous pemphigoid (BP).

**Case presentation:**

A 75-year-old male presented with knee pain that had not improved despite 5 years of medication. We performed TKA of the left knee, placing a Stryker posteriorly stabilized prosthesis. Three days later, blisters developed near the buttocks and thighs and, on day 10 after surgery, around the operative site. A skin biopsy revealed BP. Commencing on day 14 after surgery, prednisolone 10 mg was administered twice daily. The symptoms improved by 3 weeks after surgery and were healed at 4 months. After 1 year, we performed TKA of the right knee. On day 2 after surgery, as formerly, blisters developed on the buttocks and an immediate biopsy revealed BP. Commencing on day 3 after surgery, prednisolone 10 mg was administered twice daily. On day 10 after surgery, the blisters on the buttocks had improved and no blisters were observed at the surgical site. All symptoms had resolved by 2.5 months after surgery.

**Conclusions:**

After TKA surgery, generalized BP may develop, diagnosed via skin biopsy. A quick diagnosis is important because early treatment can prevent symptom progression and shorten treatment.

## Background

Total knee replacement arthroplasty (TKA) is frequently performed in South Korea (approximately 70,000 cases in 2013 alone) [1]. Simple swelling-associated blistering around the periphery of the operative wound is a well-known adverse effect; the blisters may be painful, prevent wound-healing, and increase the risk of infection [2]. Bullous pemphigoid (BP) is an autoimmune blistering disease, predominantly of the elderly [3]. The etiology is complex, featuring predisposing genetic factors, medication use, and viral infection. Several cases developing after trauma and surgery have been reported [3–12]. However, BP after arthroplasty such as TKA has been rarely reported [12]. Only three cases of generalized BP after surgery have been described [3, 5, 12]. We diagnosed and treated a patient who rapidly developed generalized BP after TKA of the left knee. Then, 1 year later, after right knee TKA, similar symptoms developed; we quickly diagnosed and treated generalized BP. He recovered faster than before. This paper is the first report of repeated generalized BP after TKA in both knees, and shows that rapid diagnosis and treatment may be important for BP after TKA.

## Case presentation

**Fig. 1:**
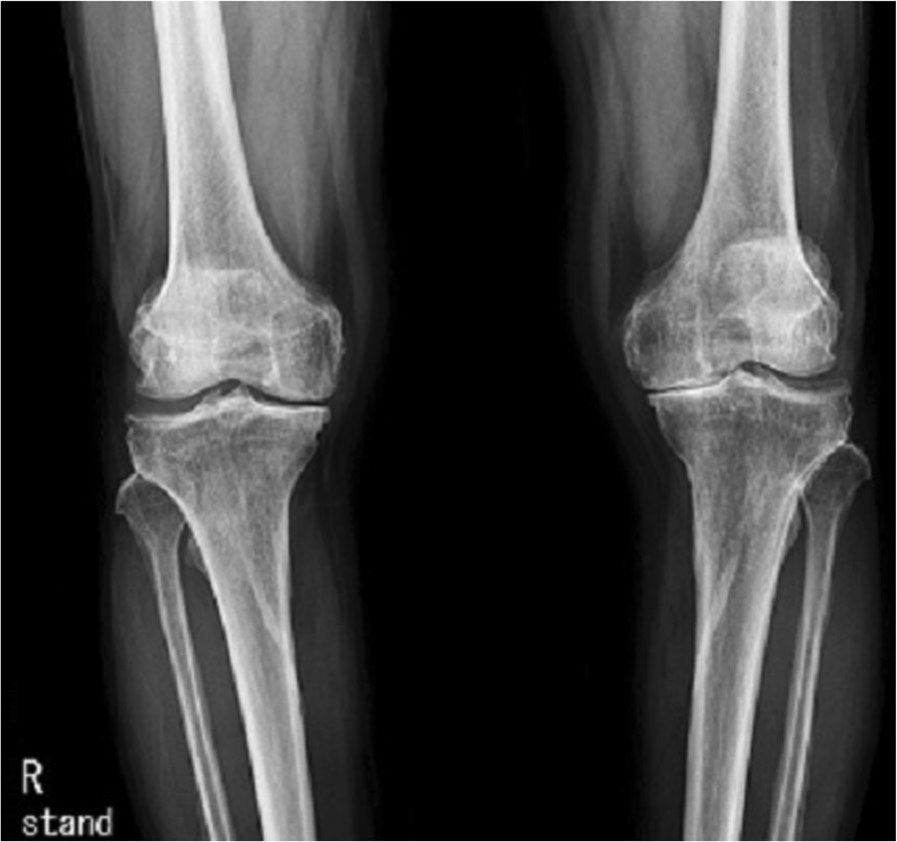
A plain radiograph reveals severe osteoarthritis with joint-space narrowing, osteophyte formation, and sclerosis

**Fig. 2:**
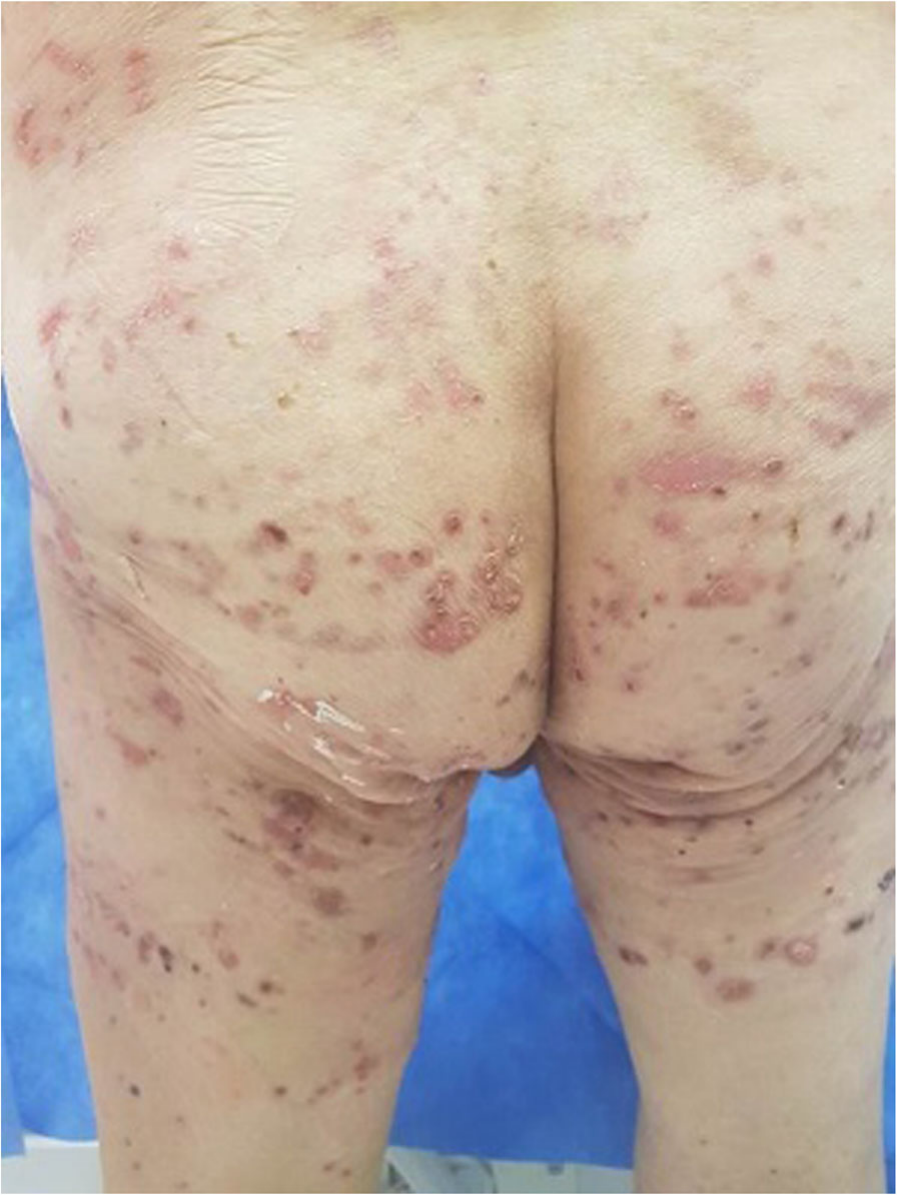
A photograph taken on day 3 after surgery shows blisters near the buttocks and thighs

**Fig. 3:**
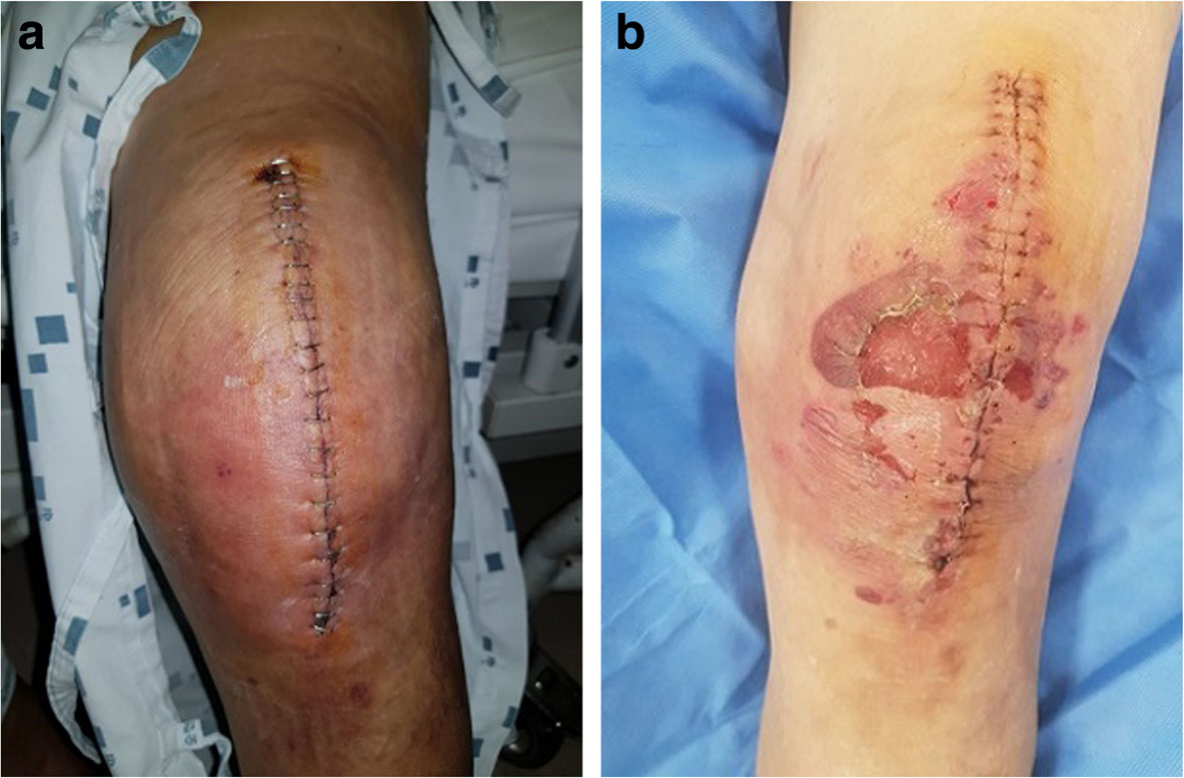
**a** A photograph taken on day 6 after surgery shows erythema around the periphery of the operative site. **b**. A photograph taken on day 10 after surgery shows blisters around the operative site

**Fig. 4:**
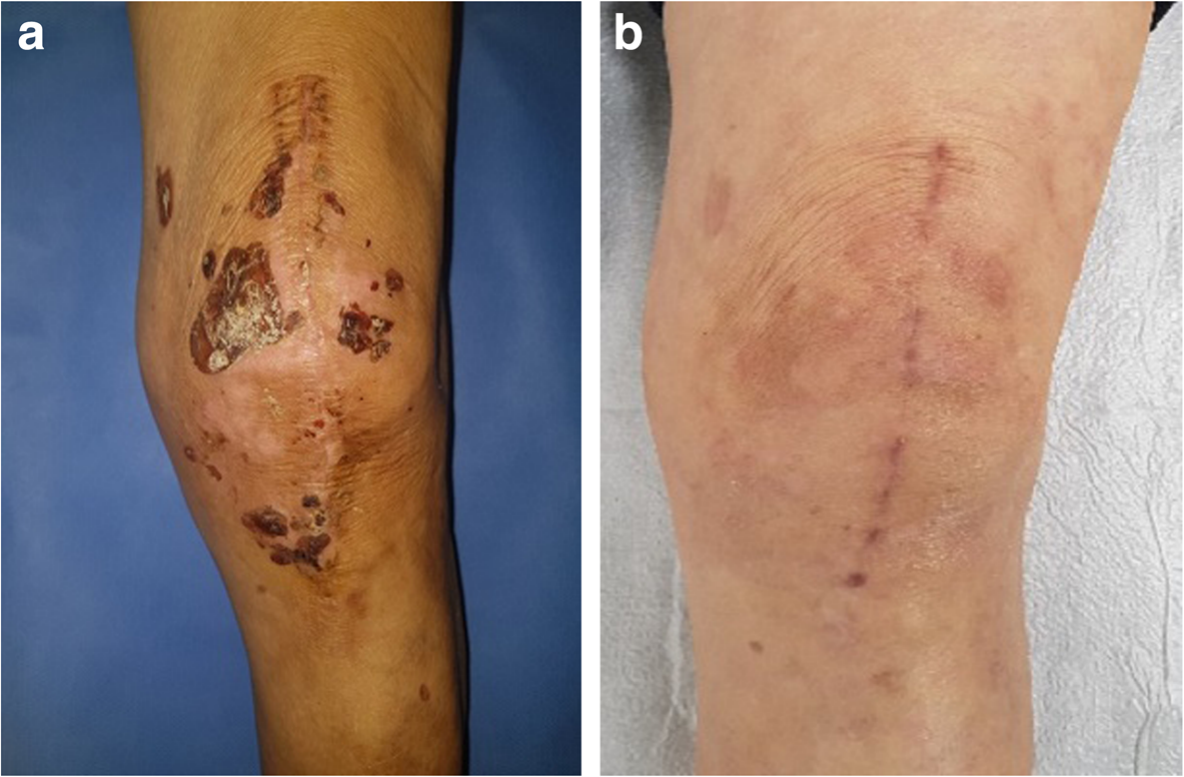
**a** A photograph taken 3 weeks after surgery shows symptom improvement around the operative site. **b**. A photograph taken 4 months after surgery shows complete healing of BP

A 75-year-old male presented with knee pain that had not improved despite 5 years of medication. Plain radiography revealed osteoarthritis of Kellgren-Lawrence grade 4 (Fig. 1). The patient had hypertension and diabetes mellitus for which he was prescribed valsartan, linagliptin, simvastatin, metformin, amlodipine, and glimepiride. Laboratory tests yielded no specific findings. He had no history of allergy. There was a history of surgery for a medial malleolus fracture under spinal anesthesia several years ago and there were no specific problems at the time. Under spinal anesthesia, TKA of the left knee was performed with placement of a Stryker posteriorly stabilized prosthesis. Three days postoperatively, blisters developed near the buttocks and thighs (Fig. 2). Under suspicion of irritant contact dermatitis, antihistamines (Twolion, Xyzal) were prescribed and methylprednisolone aceponate 0.1% (w/w) ointment (Advantan) applied in a dressing. On day 6 after surgery, erythema developed around the periphery of the operative site (Fig. 3a) and, on day 10 after surgery, blisters developed in the same region (Fig. 3b), persisted on the buttocks and thighs, and spread to the right forearm. A buttock blister was biopsied. The basement membrane was strongly positive for IgG, with some IgA and complement 3; we thus diagnosed BP. In order to suppress blister formation and prevent spreading to other areas, through consultation with a dermatologist, commencing on day 14 after surgery, prednisolone 10 mg was administered twice daily and maintained for 2 weeks. The symptoms improved 3 weeks after surgery (Fig. 4a) and healed by 4 months (Fig. 4b).

**Fig. 5:**
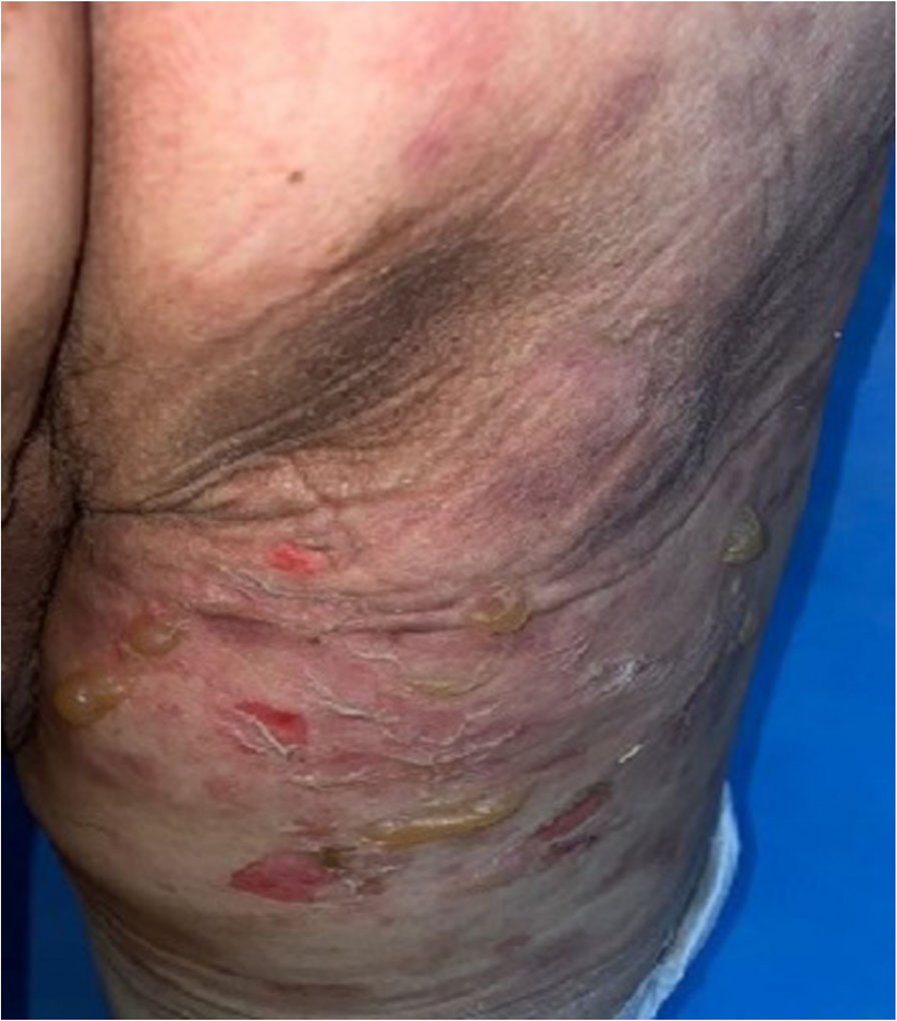
A photograph taken on day 2 after surgery on the right knee showing blisters near the buttocks and thighs

**Fig. 6:**
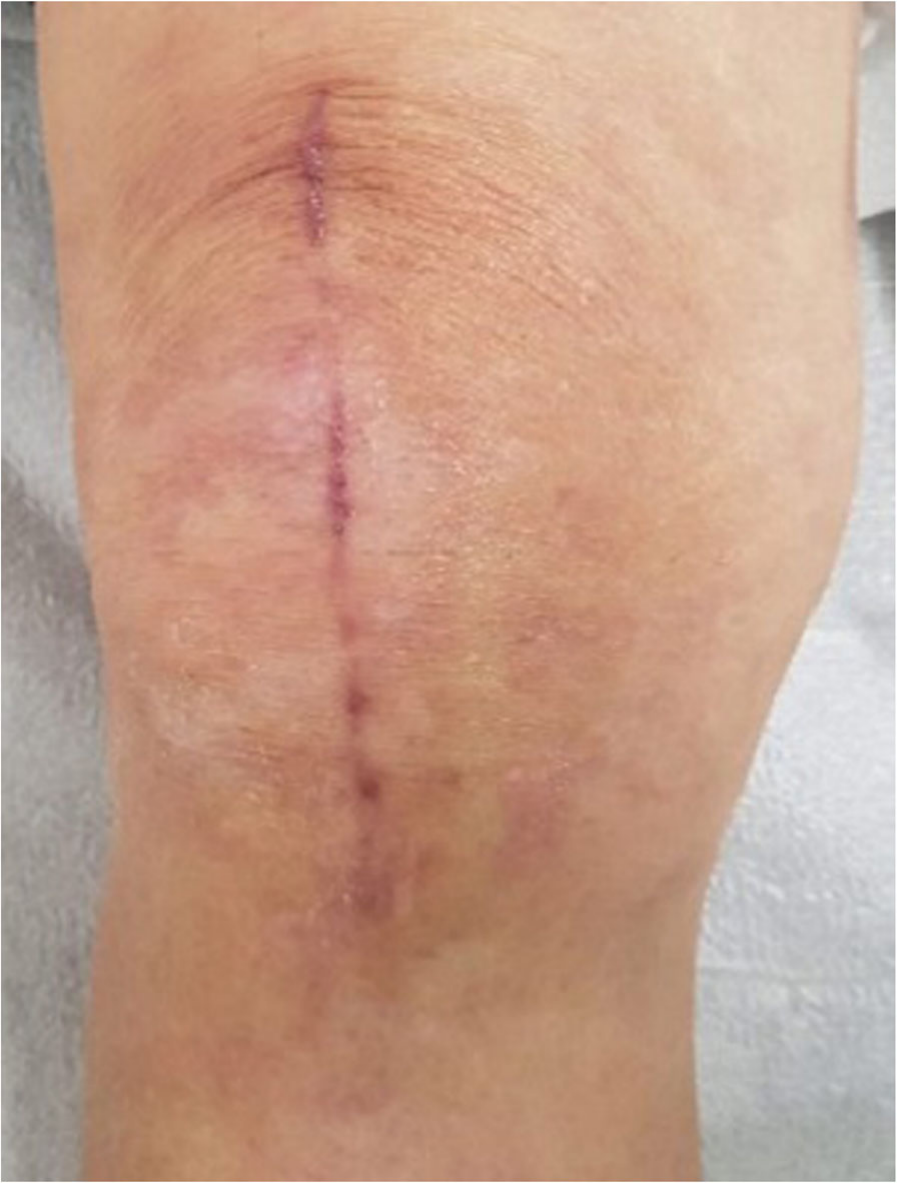
A photograph taken 2.5 months after surgery on the right knee shows complete healing of BP

After 1 year, TKA of the right knee was performed under spinal anesthesia because pain persisted despite medication. He remained on the medications described above; a pre-surgery blood test was normal. On day 2 after surgery, as before, blisters developed on the buttocks (Fig. 5). An immediate biopsy revealed BP. Commencing on day 3 after surgery, prednisolone 10 mg was administered twice daily and maintained for 2 weeks. On day 10 after surgery, the blisters on the buttocks improved and none were observed at the surgical site. All symptoms had disappeared by 2.5 months after surgery (Fig. 6).

## Discussion and conclusion

BP may be generalized or localized; the latter type may develop after surgery, wounding, or trauma, as well as within burns, in regions of cellulitis, after radiation therapy and psoralen ultraviolet A therapy, and during treatment with topical fluorouracil. Generalized BP is generally caused by drugs such as furosemide. However, several reports on generalized BP developing after surgery have appeared [3, 5, 12]. The blisters appeared on the periphery of surgical wounds and then spread to other areas of the body. We report a case of generalized BP that developed first on the buttocks and thighs (remote from the surgical site) 2–3 days after surgery. This is the first case in which such a phenomenon re-occurred (1 year later in our case).

BP presents as tense bullae on either erythematous or apparently normal skin; a lesional biopsy reveals a subepidermal blister and a dermal inflammatory infiltrate consisting primarily of neutrophils and eosinophils. Diagnosis is confirmed when direct or indirect immunofluorescence tests reveal IgG and/or C3 deposition along the basement membrane, or circulating IgG autoantibody. The salt-split skin technique or electron microscopy exclude epidermolysis bullosa acquisita [5].

Truss et al. [12] reported BP that progressed rapidly after TKA, and emphasized the need for a differential diagnosis that excluded simple swelling-associated blistering, allergic contact dermatitis, and bullous impetigo. In the cited case, blisters first developed around the periphery of the surgical site and, after 5 months, spread to other bodily areas. It was not possible to perform an early skin biopsy because of a concern the biopsy might trigger surgical site infection. In our case, blisters first developed on the buttocks, and then progressed rapidly to the wound region. Buttock lesional biopsy was possible without any risk of surgical site infection; diagnosis was relatively rapid.

BP blistering reflects the binding of circulating IgG (and occasionally IgA) antibodies to various BP antigens (principally BP230 or BP180) associated with hemidesmosomes within dermo-epidermal junctions. The complement pathway becomes activated, associated with leucocyte migration, mast cell degranulation, and release of inflammatory cytokines. Polymorphic cells then infiltrate the dermis and release lysosomal enzymes that disrupt the dermo-epidermal junction and trigger subepidermal blister formation [13].

Localized BP developing after trauma or surgery has given rise to the concept of an “immunocompromised district”. Tissue damage interferes with local immune and cutaneous processes, rendering the development of secondary conditions such as BP possible [14]. However, this does not explain the generalized BP that we encountered. Although it is not possible to completely exclude all triggering factors, the patient had no previous allergy history and there was no problem in the surgery previously under spinal anesthesia. In addition, there was no use of medication that was not used before TKA, and there was no change in the patient’s surrounding environment before and after surgery. The fact that generalized BP occurred twice suggests a link with surgery.

The most common BP medications are anti-inflammatory agents and immune suppressants [15]. Systemic corticosteroids are the best-established treatment. Depending on symptom severity, prednisolone (30 to 70 mg daily) is the recommended initial drug [15]. We consulted a dermatologist; we prescribed 20 mg daily for 2 weeks. After surgery on the left knee, prednisolone commenced 2 weeks after surgery; after right knee surgery, treatment began 3 days later. No blister developed around the wound after the second surgery; rapid diagnosis and steroid prescription halted disease progression.

BP blisters rarely develop after TKA. Most reports described localized BP around the periphery of the surgical site; generalized BP was very rare. In our case, generalized BP developed after left knee TKA and was treated via an oral steroid. One year later, after right knee TKA, generalized BP developed again, and was (of course) diagnosed and treated very quickly; recovery was rapid. Although generalization is inappropriate given that we describe only one case, we suggest that early treatment can shorten the treatment period. Therefore, if blistering appears after TKA, BP should be differentiated and perform an immediate skin biopsy for early diagnosis and treatment even if blistering is systemic. Blisters around the surgical site may be biopsied if there is no sign of infection. Blisters developing in a patient who evidenced BP after prior surgery must be biopsied.

## Data Availability

Not applicable.

## Abbreviations

TKA: Total knee replacement arthroplasty
BP: Bullous pemphigoid
